# Circulating Levels of sFlt1 Splice Variants as Predictive Markers for the Development of Preeclampsia

**DOI:** 10.3390/ijms160612436

**Published:** 2015-06-02

**Authors:** Colby A. Souders, Sharon E. Maynard, Jing Yan, Yang Wang, Naomi K. Boatright, Jessica Sedan, David Balyozian, Peter S. Cheslock, Deborah C. Molrine, Tiffany A. Moore Simas

**Affiliations:** 1 MassBiologics of the University of Massachusetts Medical School, Boston, MA 02126, USA; E-Mails: Yang.Wang@UMassMed.edu (Y.W.); nboatwpi@gmail.com (N.K.B.); Jessica.Sedan@UMassMed.edu (J.S.); David.Balyozian@UMassMed.edu (D.B.); Peter.Cheslock@UMassMed.edu (P.S.C.); Deborah.Molrine@UMassMed.edu (D.C.M.); 2 Department of Medicine, Division of Nephrology, Lehigh Valley Health Network, University of South Florida Morsani College of Medicine, Allentown, PA 18105, USA; E-Mail: sharon_e.maynard@lvhn.org; 3 Department of Biochemistry and Molecular Pharmacology, University of Massachusetts Medical School, Worcester, MA 01655, USA; E-Mail: jyan1013@gmail.com; 4 Department of Obstetrics and Gynecology, University of Massachusetts Medical School/UMass Memorial Health Care, Worcester, MA 01605, USA; E-Mail: TiffanyA.MooreSimas@umassmemorial.org; 5 Department of Pediatrics, University of Massachusetts Medical School, Worcester, MA 01655, USA

**Keywords:** preeclampsia, soluble fms-like tyrosine kinase 1 (sFlt1), splice variants, isoforms, monoclonal antibody (mAb), diagnostic

## Abstract

Angiogenic biomarkers, including soluble fms-like tyrosine kinase 1 (sFlt1), are thought to be predictors of preeclampsia onset; however, improvement is needed before a widespread diagnostic test can be utilized. Here we describe the development and use of diagnostic monoclonal antibodies specific to the two main splice variants of sFlt1, sFlt1-1 and sFlt1-14. These antibodies were selected for their sensitivity and specificity to their respective sFlt1 isoform in a capture ELISA format. Data from this pilot study suggest that sFlt1-1 may be more predictive of preeclampsia than total sFlt1. It may be possible to improve current diagnostic platforms if more specific antibodies are utilized.

## 1. Introduction

Preeclampsia is a multi-system disorder characterized by new onset of hypertension and proteinuria after 20 weeks of gestation and affects 3%–8% of pregnancies [1,2]. Eclampsia, the development of grand mal seizures, is estimated to occur in 1%–2% of women with preeclampsia [3]. In the United States, preeclampsia/eclampsia is one of the leading causes of maternal death, while globally 10%–15% of maternal deaths related directly to obstetric complications are associated with preeclampsia/eclampsia [4,5]. Several risk factors for preeclampsia have been identified [3]; for women with chronic hypertension and/or pre-existing renal disease, superimposed preeclampsia can be difficult to diagnose [6,7,8,9].

Investigations into underlying pathophysiological mechanisms of this disorder have focused on angiogenic factors and are reviewed in detail elsewhere [10,11,12]. One theory proposes that dysregulation of angiostatic processes, specifically an excess of circulating soluble fms-like tyrosine kinase 1 (sFlt1), interferes with vascular endothelial growth factor (VEGF) and placental growth factor (PlGF) binding to, and interacting with, cell surface receptors such as Flt1, thus promoting the mitogenesis of endothelial cells [13,14,15,16,17,18]. Regulation of such signaling pathways appears to be important for appropriate angiogenesis in the developing placenta and provides an adaptive explanation for the role of sFlt1 in normal pregnancy. It also suggests how an excess of the sFlt1 protein might result in abnormal development of the placenta and impact other maternal organs where the maintenance and regulation of endothelial barriers are important.

Alternative splicing of the *VEGFR-1* (*FLT1*) gene produces the soluble form [19] and the role of sFlt1 in preeclampsia is further complicated by the existence of multiple splice variants of sFlt1. At least two splice variants, sFlt1-1 (also known as sFlt1_v1 [20] and sFlt1-i13 [21]) and sFlt1-14 (also known as sFlt1_v2 [20] or sFlt1-e15a [21]), are differentially expressed and distributed in human tissues [20,21,22,23,24]. Based on mRNA expression, the sFlt1-14 variant is highly placenta-specific, whereas sFlt1-1 is expressed in several tissues [23]. sFlt1-14 levels have been shown to increase dramatically in women with preeclampsia, suggesting that the sFlt1-14 variant may be a significant factor in the pathogenesis of preeclampsia [22,25]. A large change in circulating concentrations of sFlt1 proteins can be detected prior to clinically evident disease [15,26,27,28,29] and measurement of specific sFlt1 splice variants over time might improve methods to predict the development of preeclampsia [23,30]. Biomarkers for pregnant women with risk factors, such as chronic hypertension and/or diabetes, may be especially useful as clinical diagnosis guidelines for preeclampsia are highly reliant on blood pressure and proteinuria thresholds which may already be exceeded in the context of these respective underlying co-morbidities. Monitoring concentration changes of these variants in earlier gestational windows may augment and improve clinical diagnostic precision in the presence of co-morbidities and thus impact clinical management.

Here we report the development, characterization and evaluation of sFlt1 splice variant-specific monoclonal antibodies (mAbs) to measure levels of sFlt1 isoforms in serum samples collected prospectively from pregnant women who develop preeclampsia and matched controls. The objective of this laboratory-based study was three-fold: (1) To develop sFlt1 isoform-specific monoclonal antibodies; (2) To assess the relative abundance of each sFlt1 isoform within pre-defined gestational windows in singleton pregnancies with a normal outcome as compared to those that progress to preeclampsia; and (3) To determine if sFlt1 isoforms are stronger predictive biomarkers of preeclampsia as compared to total sFlt1 measurement, particularly in women with known risk factors for this disorder.

## 2. Results

### 2.1. Generation of Total Soluble Fms-Like Tyrosine Kinase 1 (sFlt1) and Isoform-Specific Monoclonal Antibodies

**Figure 1:**
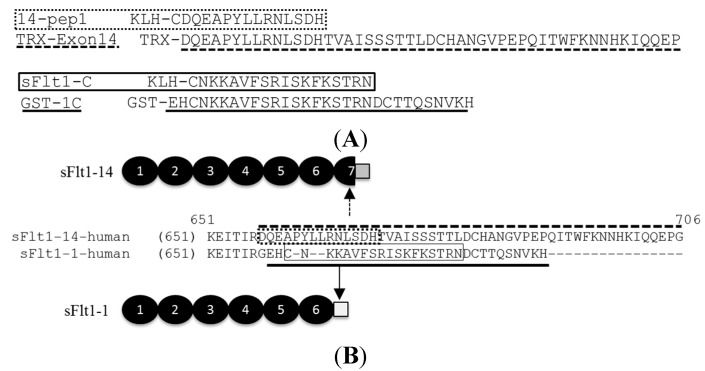
*C*-terminal peptide sequences used for generation of mouse mAbs specific to sFlt1-1 (solid line) and sFlt1-14 (dashed line). (**A**) A keyhole limpet hemocyanin (KLH) fusion protein preceded each sFlt1 peptide antigen used for immunizations while a Thioredoxin (TRX) or glutathione *S*-transferase (GST) tag preceded the sFlt1-14 and sFlt1-1 peptides, respectively, used for screening ELISAs. Full-length sFlt1-1 containing a *C*-terminal his tag was used to generate all other mouse mAbs and HuMAbs that recognize total sFlt1; (**B**) Schematic of sFlt1-14 and sFlt1-1 proteins identifying the unique epitopes antibodies were directed against.

**Figure 2:**
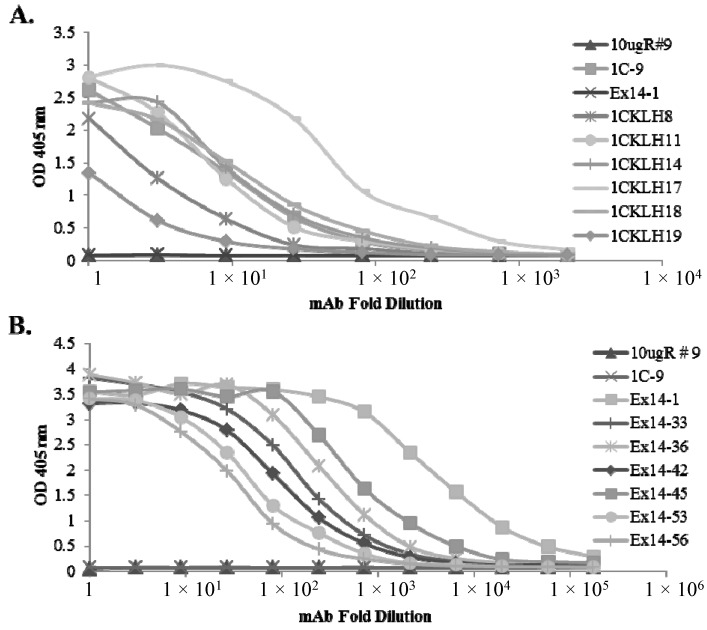
Hybridoma cultures were screened by ELISA against (**A**) GST-1C or (**B**) TRX-Exon14 to isolate sFlt1-1 or sFlt1-14 specific mAbs, respectively. 1CKLH18 and Ex14-1 were selected for their favorable binding properties as isoform-specific mAbs, while the total sFlt1-specific mAb, 10ugR#9, which binds a shared domain of the full-length protein, was included as a negative control.

**Figure 3:**
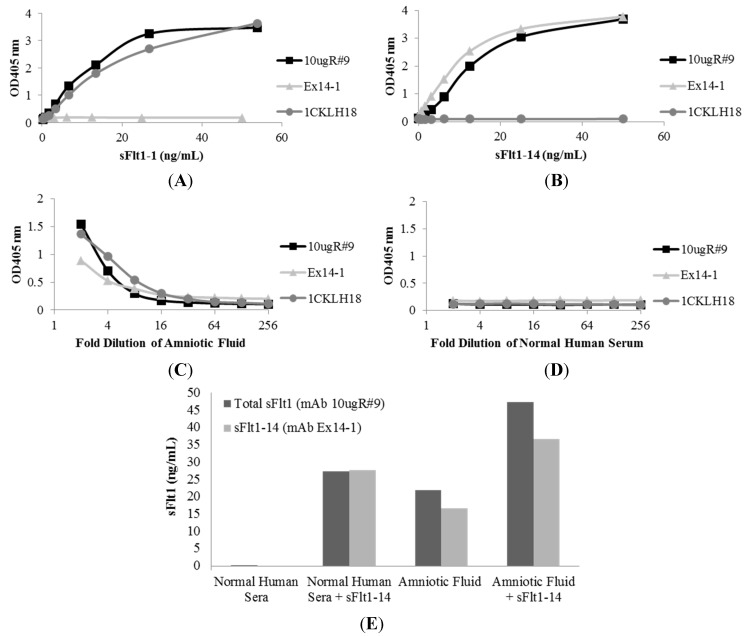
Mouse mAbs were specific for sFlt1 splice variants as measured by capture ELISA. Total sFlt1-specific mAb, 10ugR#9, recognized both recombinant sFlt1-1 (**A**) and sFlt1-14 (**B**) splice variants with similar sensitivity; however, mAb 1CKLH18 only detected sFlt1-1 while mAb Ex14-1 only detected sFlt1-14. In addition, each mAb specifically recognized endogenous sFlt1 present in amniotic fluid (**C**), while biological fluid that does not contain sFlt1 (normal human serum) was not detected (**D**). To assess interference of quantitation in biological fluids (**E**), 25 ng/mL of recombinant sFlt1-14 was spiked into normal human sera or amniotic fluid.

CD-1 wild type mice or HuMAb mice were immunized with full-length sFlt1-1, sFlt1-C or 14-pep1 (Figure 1). Sera responses and subsequent hybridoma cultures following splenic fusion were monitored by ELISA against full-length sFlt1-1 and sFlt1-14 (Figure 1B) or isoform-specific peptides GST-1C and TRX-Exon14 (Figure 1A). Of the 359 total positive murine clones, several had specific activity only to sFlt1-1 (Figure 2A) or sFlt1-14 (Figure 2B), as shown by ELISA. Mouse mAb 10ugR#9 was selected for its ability to bind both full length sFlt1-1 and sFlt1-14 (Figure 3A,B), but not the isoform-specific peptides GST-1C or TRX-Exon14 (Figure 2). Of the isoform-specific clones shown in Figure 2, mouse mAb 1CKLH18 and Ex14-1 were selected for their specific binding properties to sFlt1-1 and sFlt1-14, respectively (Figure 3A,B).

### 2.2. Characterization of sFlt1-Specific Monoclonal Antibodies by ELISA

A capture ELISA was developed to measure the concentration of total sFlt1 and its isoforms, sFlt1-1 and sFlt1-14, in biological fluid. A human anti-total sFlt1 mAb with binding properties similar to mouse mAb 10ugR#9, but that did not compete with 10ugR#9, was used as a coating antibody. Using the human anti-total sFlt1 mAb to capture all sFlt1 isoforms, followed by mouse mAbs 10ugR#9, 1CKLH18 or Ex14-1 to detect bound sFlt1, allowed for the efficient capture of total sFlt1 or individual isoforms in biological fluid without high background or interference (Figure 3). The total sFlt1-specific detection mAb, 10ugR#9, recognized both recombinant human sFlt1-1 (Figure 3A) and sFlt1-14 (Figure 3B) expressed and purified from CHO cells at comparable sensitivities. The sFlt1-1-specific mAb, 1CKLH18, detected recombinant human sFlt1-1, but not sFlt1-14. Conversely, the sFlt1-14-specific mAb, Ex14-1, detected recombinant human sFlt1-14, but not sFlt1-1 (Figure 3A,B). Each mAb detected sFlt1 isoforms in human amniotic fluid (Figure 3C) and undetectable levels of these sFlt1 isoforms were observed in normal human sera from men or non-pregnant women (Figure 3D).

Using human recombinant sFlt1-14 to generate standard curves, sFlt1-14 concentration in amniotic fluid was quantified (Figure 3E). As expected, this protein was not detected in normal human serum (<0.02 ng/mL) and when 25 ng/mL of human recombinant sFlt1-14 was added to the sera sample, both mAbs 10ugR#9 and Ex14-1 quantified the spiked sFlt1-14 at 27.32 and 27.77 ng/mL, respectively (Figure 3E), indicating the mAbs were specific for total sFlt1 or its isoform and did not recognize additional proteins in biological fluid.

Quantification in amniotic fluid showed levels of total sFlt1 to be 21.87 ng/mL and sFlt1-14 at 16.64 ng/mL (Figure 3E). Similar to sera samples, 25 ng/mL of sFlt1-14 added to amniotic fluid was detected, as the total levels measured were approximately equal to the sum of spiked sFlt1-14 plus the endogenous levels measured (47.4 ng/mL for 10ugR#9 and 36.63 ng/mL for Ex14-1; Figure 3E). These results demonstrate that the mAbs were able to specifically recognize sFlt1 isoforms in human biological samples without interference in quantitation.

### 2.3. Specificity of Anti-sFlt1 Monoclonal Antibodies by Western Blot

**Figure 4:**
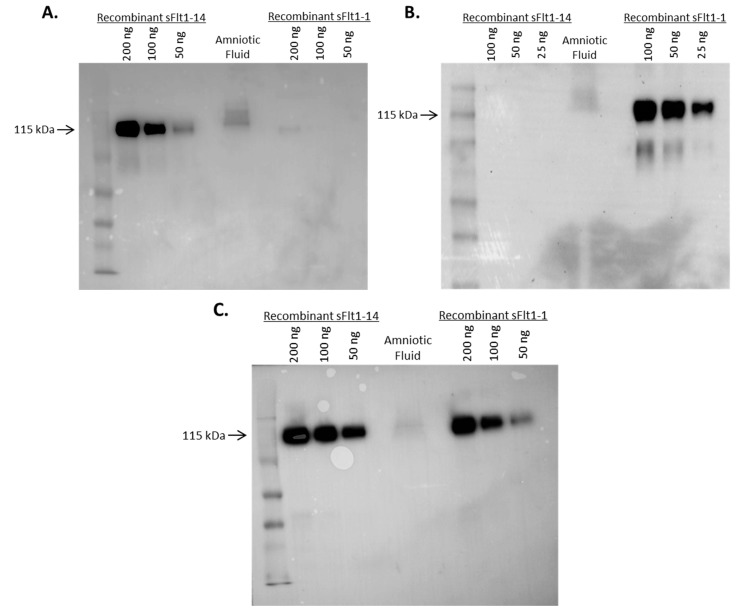
Mouse mAbs Ex14-1 (**A**) and 1CKLH18 (**B**) specifically detected endogenous sFlt1 isoforms from amniotic fluid on a Western blot. Each mAb recognized a single protein of the expected molecular weight (~115 kDa) when compared to recombinant standards. Included as a positive control (**C**) is a commercially available mAb (Sigma Cat.#V4262) that recognized total sFlt1.

Specificity of mAbs to sFlt1-14 and sFlt1-1 was further investigated by Western blot analysis on human amniotic fluid (Figure 4). Both Ex14-1 (Figure 4A) and 1CKLH18 (Figure 4B) recognized a single protein in amniotic fluid at the expected molecular weight (~115 kDa) that corresponds to recombinant human sFlt1-14 and sFlt1-1, respectively. In addition, both mouse mAbs specifically recognized their recombinant sFlt1 isoform standards by Western blot. These data confirm the ELISA results suggesting the mouse antibodies were specific for their sFlt1 isoforms and recognized their endogenous sFlt1 isoform in biological fluid.

### 2.4. Affinity of Anti-sFlt1 Monoclonal Antibodies

The *K*_D_ of mAb Ex14-1 and 1CKLH18 was measured to be 3.95 × 10^−8^ and 4.30 × 10^−9^ M, respectively, by Octet analysis. In comparison, the *K*_D_ of mAb 10ugR#9 was 5.4 × 10^−9^ M and the human mAb used as a capture antibody was 2.57 × 10^−9^ M. The nanomolar or near-nanomolar affinities of these mAbs made them sensitive reagents for a diagnostic assay with quantitative capabilities.

**Table 1:** Demographic Characteristics of Study Subjects.

Demographic Characteristics	Control Subjects (*n* = 137) *	Subjects with Preeclampsia (*n* = 15) *
Maternal age (years)	31.3 (5.1)	32.8 (5.6)
Gravity	2.4 (1.5)	2.1 (1.3)
Essential Nulliparity	43 (31.4)	7 (46.7)
Body mass index (kg/m^2^):		
Underweight (BMI < 18.5)	1 (0.7)	0 (0.0)
Normal weight (BMI 18.5–24.9)	23 (16.8)	1 (6.7)
Overweight (BMI 25.0–29.9)	43 (31.4)	6 (40.0)
Obese (BMI ≥ 30.0)	70 (51.1)	8 (53.3)
Systolic BP at enrollment (mmHg)	117.9 (14.4)	123.1 (10.7)
Diastolic BP at enrollment (mmHg)	71.7 (10.2)	73.4 (15.9)
Anti-hypertensive medications at enrollment	19 (13.9)	5 (33.3) (exact *p*-value = 0.0639)
Smoking status:		
Current smoker	12 (8.8)	0 (0.0)
Lives with smoker	18 (13.1)	3 (20.0)
Chronic hypertension †	23 (16.8)	6 (40.0)
Pregestational diabetes (DM1 or DM2) †	16 (11.7)	5 (33.3)
History of prior preeclampsia	30 (21.9)	1 (6.7)
Obese (BMI ≥ 30) nullipara	28 (20.4)	4 (26.7)
Any severe preeclampsia	0 (0.0)	12 (80.0)

preeclampsia (PE), chronic hypertension (cHTN), diabetes mellitus (DM), kilograms/meter^2^ (kg/m^2^), body mass index (BMI), blood pressure (BP), millimeters mercury (mmHg); * Data are mean (±standard deviation) or number (%); † *p* < 0.05 for comparisons between control and preeclampsia cohorts.

### 2.5. Measurement of sFlt1 Variants in the Sera of Pregnant Women

**Figure 5:**
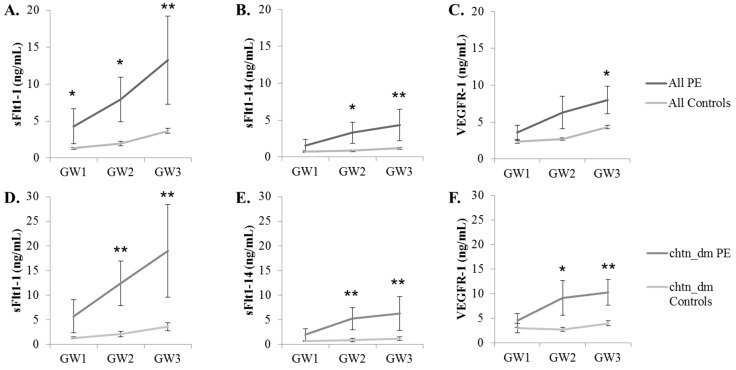
sFlt1 isoform and VEGFR-1 quantitation from serum samples at three gestational windows (GW) during pregnancy. (**A**) sFlt1-1, (**B**) sFlt1-14 and (**C**) VEGFR-1 levels from all women included in the study and (**D–F**, respectively) a subset from women included in A–C diagnosed with chronic hypertension and/or diabetes mellitus (chtn_dm) are reported as the mean biomarker level ± SEM. * *p* ≤ 0.05; ** *p* ≤ 0.01.

Longitudinal serum samples prospectively collected under IRB approval from pregnant women [31,32] included women with or without risk factors for preeclampsia. Complete cohort demographics are illustrated in Table 1. Samples were analyzed for their concentration of sFlt1 splice variants using a capture ELISA format with mAbs Ex14-1 and 1CKLH18 to detect sFlt1-14 and sFlt1-1 splice variants, respectively. These results were compared to the concentration of total sFlt1 levels (VEGFR-1) that had been measured previously for each sample using a Quantikine ELISA Kit. The concentrations of serum sFlt1-1, sFlt1-14 and total sFlt1 (VEGFR-1) were compared between women with a singleton gestation who developed preeclampsia (PE) to those women who did not develop preeclampsia (Control) (Figure 5A–C) for three gestational age windows (GW): 21–27.99 weeks (GW1); 28–31.99 weeks (GW2): and >32 weeks (GW3). Concentrations of the sFlt1-1 variant were significantly higher in women with preeclampsia (*n* = 13) compared to controls (*n* = 124) for the earliest gestational window (GW1). In addition, sFlt1-1 and sFlt1-14 measurements were both significantly higher in women with preeclampsia (*n* = 12) compared to controls (*n* = 115) in GW2. VEGFR-1 measurements were not significantly different between women with preeclampsia as compared to controls for GW1 or GW2; however, VEGFR-1, sFlt1-1 and sFlt1-14 concentrations were significantly different between women with preeclampsia (*n* = 10) compared to control women (*n* = 121) for GW3.

A logistic regression analysis for all women included in the study was performed to examine if any of the risk factors were independently associated with the development of preeclampsia. The presence of pre-existing chronic hypertension and/or diabetes mellitus was associated with an increased risk of developing preeclampsia (*p* = 0.0123). Therefore, comparisons of VEGFR-1 and both splice variants were performed for the subset of women with pre-existing chronic hypertension and/or diabetes mellitus who developed preeclampsia (chtn_dm PE; *n* = 9) or not (chtn_dm Controls; *n* = 29) (Figure 5D–F). For GW2 and GW3, VEGFR-1, sFlt1-1 and sFlt1-14 were significantly higher in those women who developed preeclampsia compared to controls with similar co-morbidities. Statistical differences for sFlt1-1 and sFlt1-14 were greater at GW2 when compared to VEGFR-1.

**Figure 6:**
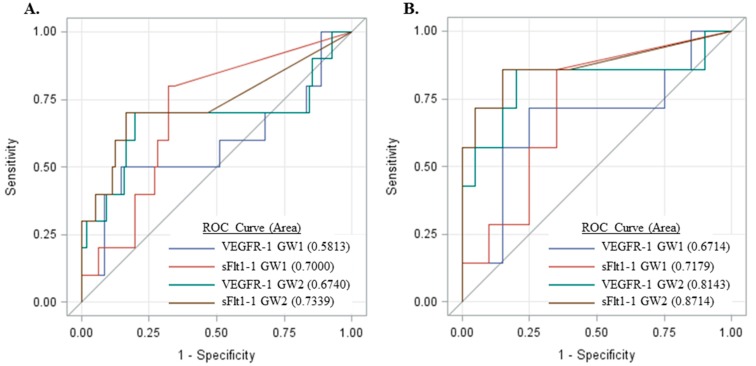
Receiver operator curves generated from the sensitivity and specificity of sFlt1-1 and VEGFR-1 preeclampsia predictions at gestational windows 1 and 2 in (**A**) all samples measured and (**B**) a high-risk subset of these women with chronic hypertension and/or diabetes mellitus.

These results suggest measurement of sFlt1 isoforms, particularly sFlt1-1, may be more predictive of preeclampsia as compared to VEGFR-1 (total sFlt1). Thus, receiver operator curves (ROC) were generated for subjects who had samples at both GW1 and GW2 time points (Figure 6). The area under the curve (AUC) for sFlt1-1 was greater as compared to VEGFR-1 for both GW1 and GW2 (Figure 6A) and, furthermore, the sFlt1-1 AUC at GW1 was comparable to that of VEGFR-1 at GW2. For subjects who developed preeclampsia, the GW1 sample was collected, on average, 10.2 weeks before preeclampsia diagnosis while collection at GW2 was a mean of 6.99 weeks prior to diagnosis, suggesting that sFlt1-1 may be as predictive as VEGFR-1 at least three weeks earlier. Similarly, the AUC is greater for sFlt1-1 compared to VEGFR-1 at both gestational windows for the subset of women with chronic hypertension and/or diabetes mellitus (Figure 6B).

## 3. Discussion

To our knowledge, this is the first detailed characterization of sFlt1 isoform-specific monoclonal antibodies. Development of the sFlt1 isoform-specific mAbs was accomplished using the carboxy-terminus peptides described in conjunction with standard immunization and hybridoma techniques. These antibodies had high affinities and could specifically recognize their appropriate isoforms from both recombinant and endogenous sources. Using the mAbs in a capture ELISA format yielded an assay with high sensitivity to quantitate the sFlt1 isoforms in human serum.

We assessed the ability of these mAbs to measure sFlt1-1 and sFlt1-14 isoforms in human serum samples prospectively collected from pregnant women and compared these results to total sFlt1 (VEGFR-1) measured using a commercial kit similar or identical to what has been used in previous studies that include sFlt1 as a predictive biomarker for preeclampsia [15,26,27,29,32,33,34,35]. Of note, the sFlt1-14 epitope used to generate the sFlt1-14-specific mAb is shared with two other sFlt1 isoforms, sFlt1_v3 and sFlt1_v4 [20]; however, these isoforms have been shown to represent a very small portion of total sFlt1 (<1% of total sFlt1 mRNA transcripts) [23].

Measurement of sFlt1 isoforms collected prospectively from pregnant women suggested sFlt1-1 is the predominant isoform in the maternal circulation, as opposed to sFlt1-14, even in women who later developed preeclampsia. This clarifies previous studies evaluating sFlt1 isoform gene expression and is in contrast to other reports that proposed sFlt1-14 is the major isoform in at-risk pregnancies [22,24,25]. This discrepancy may be due to the smaller sample size of previous studies, origin of the sample (amniotic fluid or placenta), methods of quantitation (RNA or polyclonal antibodies), study inclusion criteria (e.g., women with major risk factors for preeclampsia not included) and gestational age at time of sampling in conjunction with preeclampsia diagnosis. While our results indicate sFlt1-1 is the predominant isoform in maternal circulation prior to preeclampsia diagnosis, it is possible sFlt1-14 becomes predominant at the time of clinical diagnosis. However, this seems unlikely since serum from the third gestational window was collected on average three weeks prior to preeclampsia diagnosis and sFlt1-1 levels were still approximately three-fold higher than those of sFlt1-14. However, very few samples were collected at the time of preeclampsia diagnosis, making the point-of-diagnosis analysis difficult to assess in this study.

Previous reports investigating mRNA levels suggested that while all sFlt1 transcripts are increased in women diagnosed with preeclampsia, sFlt1-1 may be more dramatically increased in preeclamptic pregnancies as opposed to normotensive pregnancies [20,23]. Our results extend these initial reports and show that the statistically significant difference in mean serum sFlt1 concentrations at the earliest gestational window between those women who developed preeclampsia compared to those who did not suggests the sFlt1-1 isoform may be a more predictive biomarker than total sFlt1 (also defined here as VEGFR-1). Although not significant, this conclusion was further supported by a trend in greater area under the receiver operator curves (*p* = 0.0826 *vs.* VEGFR-1 at GW2) and, interestingly, could be applied to a high-risk cohort of pregnant women with hypertension and/or diabetes mellitus who were part of the study (*p* = 0.321 *vs.* VEGFR-1 at GW2). One possible explanation for the potentially improved predictability of sFlt1-1 isoform as a biomarker (as compared to total sFlt1) is the antibody specificity. The total sFlt1 assay not only measures all isoforms of sFlt1, including sFlt1-1 and sFlt1-14, but also recognizes VEGFR-1 surface receptor proteolytically cleaved from cell membranes, which can be introduced into the circulation. Evidence suggests this mechanism is a possible significant source of soluble VEGFR-1 [36,37] and if background levels of membrane-cleaved soluble VEGFR-1 are similar among women with and without preeclampsia, this may confound the actual biomarker differences attributed to preeclampsia.

One limitation of this study was the inability to optimize assay sensitivity due to the volume of available serum. Isoform-specific ELISAs were assayed using a four-fold initial dilution of serum, with the lower limit of quantitation at 1.2 ng/mL. As a result, a significant number of samples were below the lower limit of quantitation, particularly in the sFlt1-14 assay. Subsequent studies need to be done to improve sensitivity of the ELISA and quantitate sFlt1 isoform levels closer to the lower limit of detection (300 pg/mL in the present study).

In addition, the total sFlt1 detector mouse mAb, 10ugR#9, allowed us to bridge the sFlt1-1 and sFlt1-14 ELISAs and indicated absolute quantitation of sFlt1-1 and sFlt1-14 could be directly compared within a sample. The VEGFR-1 assay lacked this correlation and thus absolute VEGFR-1 quantitation cannot be directly compared to sFlt1-1 and sFlt1-14 levels, though relative differences across samples could be reliably compared. This is likely due to a combination of factors including the antibody epitopes, affinities, ELISA format and use of different recombinant standards in the isoform specific *vs.* VEGFR-1 assays and explains why absolute quantitation of both isoforms together was typically greater than that of VEGFR-1 alone.

Finally, this study was limited by the number of samples available, particularly in the subjects developing preeclampsia. Future studies should evaluate sFlt1 isoforms with larger sample sizes to confirm these findings and more precisely determine a threshold or cutoff value that maximizes sensitivity and specificity. Recent studies have highlighted the importance of sample selection to ensure reliable and representative measurements of total sFlt1 [38].

In summary, we have developed mouse monoclonal antibodies that bind to sFlt1 variants sFlt1-1 and sFlt1-14. In a laboratory-based study, these mAbs were able to detect statistically significant differences in serum concentrations of sFlt1 isoforms between women who develop preeclampsia and those who do not. We speculate that these isoform-specific biomarkers may more accurately predict preeclampsia in both low-risk and high-risk groups of pregnant women with pre-existing hypertension and diabetes mellitus, which could impact the management of their medical care [27]. Future studies that include a larger cohort of high-risk pregnant women are needed to reliably determine the optimal threshold for prediction of preeclampsia using the sFlt1-1 isoform as a biomarker.

## 4. Experimental Section

### 4.1. Chinese Hamster Ovary (CHO) Cell Expression and Purification of Full-Length sFlt1 Isoforms

Human sFlt1-1 and sFlt1-14 amino acid and protein sequences were determined from GenBank (AAC50060.1 and EU368830.1, respectively) and a codon-optimized gene encoding the sFlt1-1 or sFlt1-14 protein (Figure 1) was synthesized by Integrated DNA Technologies (IDT, Coralville, IA, USA) and the sequence confirmed. The gene was subcloned into pcDNA3.1 (Life Technologies, Carlsbad, CA, USA) in frame with a *C*-terminal 6-histidine tag. To express sFlt1 proteins, CHO cells were transiently transfected with the vector containing the gene. Briefly, cells at a density of 1 × 10^6^ cells/mL were seeded in CD-CHO media (Life Technologies) + 6 mM l-glutamine 24 h before transfection of pcDNA3.1 vector at 1 μg/mL of culture diluted in Opti-MEM (Life Technologies). Prior to transfection, linear 25 kDa polyethylenimine (PEI; Sigma, St. Louis, MO, USA) was added to DNA (1:5 ratio of DNA/PEI) and incubated at room temperature for 15 min. Cells were re-suspended in fresh media and the transfection mix was added drop-wise to each flask. Cells were maintained at 37 °C and 5% CO_2_ with shaking for seven days before culture supernatant was harvested for protein isolation. Heparin Sepharose beads (Biorad, Hercules, CA, USA) were added to culture supernatant and mixed for 3 h at room temperature with shaking. Beads were loaded onto protein purification columns (Biorad), washed and sFlt1 protein was eluted with PBS + 1 M sodium chloride, which was subsequently dialyzed in PBS using a 10 k MWCO Slide-A-Lyzer (Life Technologies). Purified sFlt1-1 and sFlt1-14 samples were quantified by absorbance at 280 nm and a standard Bradford assay. Purity and concentration were analyzed by Coomassie stained SDS-PAGE analysis, Western blot and ELISA using anti-VEFGR1 antibody (AF321; R&D Systems, Minneapolis, MN, USA).

### 4.2. Bacterial Expression of sFlt1-1 and sFlt1-14 C-Terminal Peptides

Isoform protein sequences were aligned using VectorNTI (Life Technologies) and unique *C*-terminal domains were determined by assessing sequence homology. The isoform-specific peptides, 14-pep1 (sFlt1-14) and sFlt1-C (sFlt1-1), were synthesized by New England Peptide (Gardner, MA, USA) and chemically conjugated to keyhole limpet hemocyanin (KLH) through an *N*-terminal cysteine on each peptide. In addition, bacterially codon-optimized genes encoding *C*-terminal peptides from sFlt1-1 and sFlt1-14 were synthesized by IDT. The genes were subcloned into pET32a(+) (EMD Millipore, Darmstadt, Germany) in frame with an upstream thioredoxin (TRX) and 6-histidine tag or glutathione *S*-transferase (GST) fusion protein. L21Star *Escherichia coli* cells (Life Technologies) were transformed with the vector containing the gene and grown overnight at 37 °C in Luria-Bertani broth containing 100 μg/mL ampicillin (LB-amp). The culture was diluted 1:10 in LB-amp and grown for 2.5 h at 37 °C followed by addition of 1 mM isopropyl-β-d-thiogalactopyranoside and further grown at 37 °C for 2.5 h. Bacteria were harvested by centrifugation and pellets were frozen at −20 °C. Protein was isolated using standard Ni-NTA agarose or Glutathione sepharose bead purification and dialyzed in PBS. Protein was quantified by absorbance at 280 nm and purity was analyzed by Coomassie stained SDS-PAGE.

### 4.3. Generation of Monoclonal Antibodies

To generate mAbs specific for total sFlt1, sFlt1-1 and sFlt1-14, wild type CD-1 mice (Jackson Laboratories, Bar Harbor, ME, USA) or HuMAb mice (Bristol-Myers Squibb, New York, NY, USA) were injected intraperitoneally weekly for up to 14 weeks with 10–50 μg of sFlt1-1, 14-pep1 or sFlt1-C. The antigen was administered in combination with the Sigma adjuvant system (Sigma) per manufacturer’s protocol. Mouse sera responses were monitored by ELISA to determine the appropriate time for splenic fusion, which was generally indicated by a detectable titer against the immunizing protein at >4000-fold dilution of serum. Mouse spleens were harvested and spleen cells were isolated and fused to mouse myeloma cells (P3X-AG8.653; ATCC, Manassas, VA, USA) following a standard PEG fusion protocol to generate hybridomas. Hybridoma supernatants were screened for production of antibody reactive to sFlt1-1, sFlt1-14, TRX-Exon14 and GST-1C by ELISA. Positive clonal hybridoma cell cultures were expanded for further characterization and production of mAbs. Isolation of mAbs from culture supernatant was performed using standard Protein A purification techniques.

### 4.4. Screening and Characterization of mAbs

To determine reactivity of unique antibodies with sFlt1-1 and sFlt1-14 isoforms, ELISA plates were coated with either GST-1C or TRX-Exon14, blocked with BSA and a series of dilutions of each hybridoma supernatant containing mAb (or purified mAb) was incubated on the coated plates. Bound antibody was detected with anti-mouse alkaline phosphatase secondary antibody (Jackson ImmunoResearch, West Grove, PA, USA) and the interaction developed with pNPP. Plates were read using a Molecular Devices (Sunnyvale, CA, USA) Emax plate reader at 405 nm and antibodies with the desired specificity and highest apparent affinity were selected. Total sFlt1-specific mAbs were developed using similar methods except screening by ELISA utilized full-length human recombinant sFlt1-1 and sFlt1-14 expressed and purified from CHO cells in place of isoform-specific peptides.

Affinity of antibodies was determined using an Octet QK (ForteBio, Menlo Park, CA, USA) biomolecular interaction instrument. The Octet QK performs similarly to Biacore in the measurement of antibody affinity. The Octet QK uses biosensors to assess mass increases/decreases and determine rates of association and disassociation. Anti-murine or anti-human biosensors (ForteBio) were used to capture mAb in PBS (5 μg/mL) to saturation and unbound mAb was washed away. The coated biosensors were introduced into a solution containing recombinant human sFlt1-1 or sFlt1-14, at which time *K*_on_ was determined during this binding step. The sensor was then introduced into a buffer solution and the *K*_off_ determined. Using *K*_on_ and *K*_off_ an affinity (*K*_D_) was calculated. Reference wells were included that did not contain mAb during the capture step as well as control wells that contained either irrelevant mAb during the capture step or irrelevant antigen during the binding step. All references and controls displayed negligible binding.

### 4.5. Western Blots

Briefly, human amniotic fluid (collected under an approved IRB protocol) was concentrated in a 10 MWCO iCON protein concentrator (Life Technologies) and 15 μL was mixed 1:1 with reducing sample buffer and loaded onto 12% polyacrylamide gels. As standards, human recombinant sFlt1-1 or sFlt1-14 was loaded in final quantities of 200, 100, 50 or 25 ng. After electrophoresis and transfer to nitrocellulose membranes, blots were blocked in 5% BSA and probed with Ex14-1, 1CKLH18 or a commercially available mouse anti-VEGFR1 mAb that recognizes a shared epitope with total sFlt1 (Sigma Cat.#V4262). After washing, an HRP-labeled anti-mouse secondary antibody (Jackson ImmunoResearch) was used to detect bound mAb, developed with ECL Prime detection reagent (GE Healthcare, Wilmington, DE, USA) and visualized using a Kodak (Rochester, NY, USA) Gel Logic imaging system.

### 4.6. Quantitative Capture ELISA

A capture ELISA format was utilized to quantify the total sFlt1, sFlt1-1 or sFlt1-14 concentration in solution. Briefly, high-binding ELISA plates (Sigma) were coated with 1 μg/mL of an anti-total sFlt1 human mAb overnight at 4 °C. This anti-total sFlt1 antibody was selected to provide a capture reagent that bound both sFlt1-1 and sFlt1-14 at an epitope in their homologous regions without interfering with the availability of the epitopes bound by the primary antibodies. Plates were washed in PBS + 0.1% Tween-20 and blocked in 5% BSA/PBS + 0.1% Tween-20 for one hour. Recombinant sFlt1-1 or sFlt1-14 (used as reference standards) diluted in PBS, normal human sera (SunnyLabs, Sittingbourne, UK) or amniotic fluid (collected under an approved IRB protocol), or a 1:4 starting dilution of human sera (collected under an approved IRB protocol) in PBS was serially diluted (1:2) onto ELISA plates and incubated for 1 h at room temperature on a plate shaker at 550 rpm. After washing, one of three mouse primary antibodies were added: 10ugR#9 (detects total sFlt1), Ex14-1 (detects sFlt1-14) or 1CKLH18 (detects sFlt1-1) and incubated for one hour at room temperature. Excess primary antibody was washed and either AP or HRP conjugated goat anti-mouse IgG (Jackson ImmunoResearch) was incubated in wells for 45 min at room temperature. Following extensive washing, *p*-nitrophenyl phosphate disodium salt (pNPP) at 1 mg/mL (for AP conjugates) or either 1-step Ultra TMB ELISA detector reagent (Life Technologies) or SureBlue TMB substrate (KPL, Gaithersburg, MD, USA) (for HRP conjugates) was used according to manufacturer’s instructions and absorbance at 405 nm (for pNPP) or 450 nm (for TMB after addition of 1 N HCl) was measured using an Emax plate reader with Softmax Pro 5.4.4 software (Molecular Devices).

For prospectively collected subject samples, the lower limit of detection for sFlt1-1 and sFlt1-14 was determined to be 300 pg/mL by analysis of standard curves. Thus the lower limit of quantitation was set at 1.2 ng/mL given the 4-fold initial serum dilution. For statistical analyses, samples below the lower limit of quantitation were assigned a value equal to one-half the lower limit of quantitation, *i.e.*, 0.6 ng/mL.

Total sFlt1 (VEGFR-1) measurement in prospectively collected subject samples were previously performed [31] and data kindly provided by TMS. Briefly, the Human sVEGFR1/Flt-1 Quantikine ELISA Kit (R&D Systems) was used per manufacturer protocol. Samples at a 10- or 20-fold dilution were measured in duplicate and absolute quantities were quantified based off the recombinant standard protein included in the kit.

### 4.7. Subjects and Serum Samples for Measurement of sFlt1 Isoforms

The laboratory analysis of sFlt1 isoforms as predictive biomarkers for the development of preeclampsia was performed under a University of Massachusetts Medical School IRB–approved investigational study plan. Serum samples used in this analysis had been previously obtained prospectively from pregnant women and is described in detail elsewhere [31,32]. Briefly, samples were collected at approximately three time points: (1) 21–27 weeks gestation; (2) 28–31 weeks gestation; and (3) 32–35+ weeks gestation. All samples were stored frozen until use. Subject information collected as part of the protocol included: (a) Risk cohort (low or high); (b) Maternal outcome; (c) Singleton or multiple gestation; (d) Gestational age at each blood draw; (e) Gestational age at time of preeclampsia diagnosis, if applicable; and (f) Gestational age at delivery. Women were considered at high risk for preeclampsia if they had at least one of the following factors during pregnancy: chronic hypertension; pre-gestational diabetes mellitus; obesity, preeclampsia history and/or multiple gestations. Maternal outcome was assessed at delivery and included the following choices: (1) No pregnancy induced hypertension; (2) Gestational hypertension; (3) Early preeclampsia (<34 weeks gestation); (4) Late preeclampsia (≥34 weeks gestation); and (5) Preeclampsia severity (mild *vs*. severe) [39,40,41].

### 4.8. Statistics

Comparisons of sFlt1 proteins were performed between those women who eventually developed preeclampsia and those who did not develop preeclampsia at each gestational window. Changes in levels of sFlt1-1, sFlt1-14 and VEGFR-1 (total sFlt1) over time were expressed as means ± standard error. The means within each gestational window were compared using a standard *t*-test with unequal variances. Receiver operator curves (ROC) were generated from logistic models with diagnosed preeclampsia (yes/no) as the binary outcome and, as predictors, VEGFR-1 and sFlt1-1 values in gestational window 1 (21–27 weeks) and gestational window 2 (28–31 weeks). ROCs were generated for all women and for a subset of these women with pre-existing chronic hypertension and/or diabetes. Areas under the curves (AUC) were calculated using the trapezoidal method. Although the prospective study had pre-defined weeks for each of the three gestational windows, two samples for a single woman may have been collected within the same window due to scheduling logistics, which occurred in 13 instances out of 480 total measurements (2.7%). In these cases, one of the two measurements in the window was moved to the next closest window to avoid multiple measurements within one window from the same subject. In each instance, the sample moved to another gestational window was collected less than one week outside the pre-defined gestational window parameters. Women who were diagnosed with preeclampsia prior to sample collection were not included in the predictive modeling for that, or subsequent, window(s). Multiple gestation pregnancies were excluded for these analyses, as biomarker profiles are significantly different in the presence or absence of preeclampsia compared to singletons [42]. All analyses were performed using SAS 9.3 (SAS Institute Inc., Cary, NC, USA).

## Abbreviations

total sFlt1, VEGFR-1: Soluble fms-like tyrosine kinase 1
sFlt1-1: soluble fms-like tyrosine kinase 1 isoform v1
sFlt1-14: soluble fms-like tyrosine kinase 1 isoform v2
